# *N*,*N*′-Bis(di­phenyl­meth­yl)benzene-1,4-di­amine

**DOI:** 10.1107/S1600536813033497

**Published:** 2013-12-14

**Authors:** Aeed S. Al-Fahdawi, Hussain A. Al-Kafajy, Mohamad J. Al-Jeboori, Simon J. Coles, Claire Wilson, Herman Potgieter

**Affiliations:** a Department of Chemistry, College of Science, University of Babylon, Iraq; b Department of Chemical Engineering and Chemical Technology, Imperial College London, London SW7 2AZ, England; c UK National Crystallography Service, Chemistry, Faculty of Natural and Environmental Sciences, University of Southampton, Southampton, SO17 1BJ, England; d Diamond Light Source, Harwell Science and Innovation Campus, Didcot, Oxfordshire OX11 0DE, England; e School of Research, Enterprise and Innovation, Manchester Metropolitan University, Chester Street, Manchester M1 5GD, England

## Abstract

The complete mol­ecule of the title compound, C_32_H_28_N_2_, is generated by crystallographic inversion symmetry. The dihedral angles between the central aromatic ring and the pendant adjacent rings are 61.37 (16) and 74.20 (14)°. The N—H group does not participate in hydrogen bonds and there are no aromatic π–π stacking inter­actions in the crystal.

## Related literature   

The reduction of the Schiff-base was as described in Higuchi *et al.* (2003 ▶) and Higuchi *et al.* (2000 ▶). For the use of dendrimers in the formation of new types of organic-metallic hybrid materials, see: Kim *et al.* (2005 ▶); for drug generation, see: Basavaraj *et al.* (2009 ▶). For related structures, see: Ge & Ng (2006 ▶); Yang *et al.* (2007 ▶); Xia *et al.* (2007 ▶). Data were collected and processed according to Coles & Gale (2012 ▶).
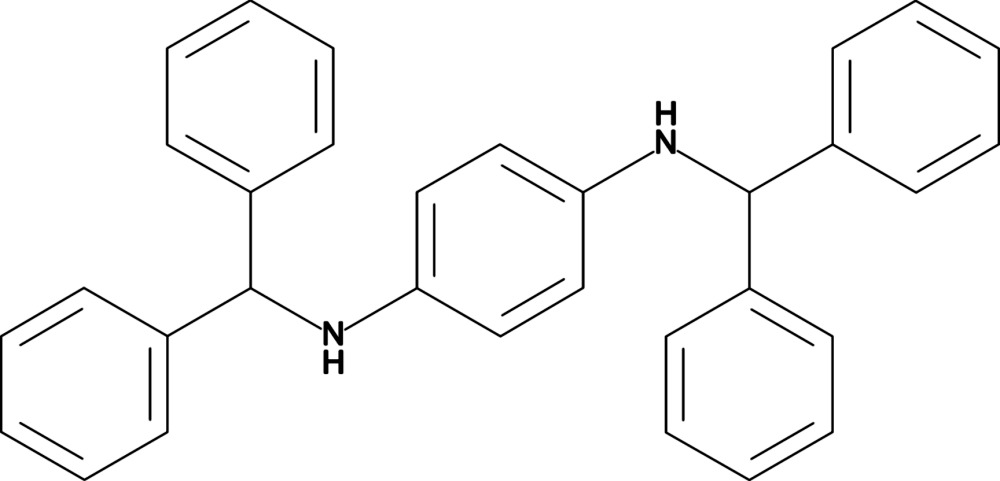


## Experimental   

### 

#### Crystal data  

C_32_H_28_N_2_
*M* *_r_* = 440.56
Monoclinic, 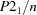

*a* = 14.784 (2) Å
*b* = 5.5853 (8) Å
*c* = 14.896 (2) Å
β = 107.914 (8)°
*V* = 1170.4 (3) Å^3^
*Z* = 2
Mo *K*α radiation
μ = 0.07 mm^−1^
*T* = 100 K
0.1 × 0.09 × 0.02 mm

#### Data collection  

Rigaku AFC12 (Right) diffractometer
Absorption correction: multi-scan (*CrystalClear-SM Expert*; Rigaku, 2012 ▶) *T*_min_ = 0.345, *T*_max_ = 1.000
10305 measured reflections
2664 independent reflections
1254 reflections with *I* > 2σ(*I*)
*R*_int_ = 0.125

#### Refinement  

*R*[*F* ^2^ > 2σ(*F* ^2^)] = 0.077
*wR*(*F* ^2^) = 0.208
*S* = 0.97
2664 reflections
155 parameters
H-atom parameters constrained
Δρ_max_ = 0.33 e Å^−3^
Δρ_min_ = −0.29 e Å^−3^

### 

Data collection: *CrystalClear-SM Expert* (Rigaku, 2012 ▶); cell refinement: *CrystalClear-SM Expert*; data reduction: *CrystalClear-SM Expert*; program(s) used to solve structure: *SHELXD* (Sheldrick, 2008 ▶); program(s) used to refine structure: *SHELXL97* (Sheldrick, 2008 ▶); molecular graphics: *OLEX2* (Dolomanov *et al.*, 2009 ▶); software used to prepare material for publication: *OLEX2*.

## Supplementary Material

Crystal structure: contains datablock(s) global, I. DOI: 10.1107/S1600536813033497/hb7158sup1.cif

Structure factors: contains datablock(s) I. DOI: 10.1107/S1600536813033497/hb7158Isup2.hkl

Supporting information file. DOI: 10.1107/S1600536813033497/hb7158Isup3.mol

Supporting information file. DOI: 10.1107/S1600536813033497/hb7158Isup4.cml

Additional supporting information:  crystallographic information; 3D view; checkCIF report

## Figures and Tables

**Fig. 1.**
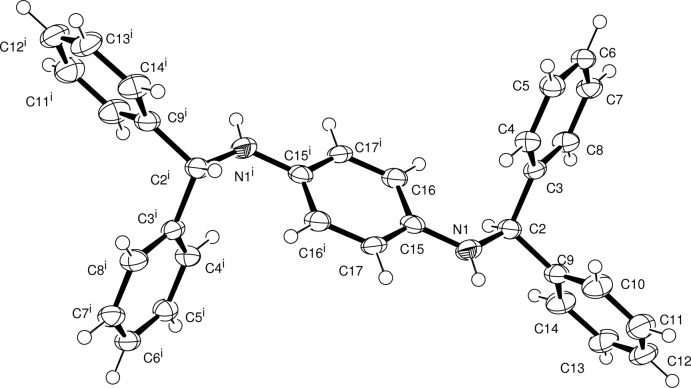
The structure of the title compound displacement ellipsoids drawn at the 50% probability level. Symmetry code: (i) 1 - x, 1 - y, -z.

## supplementary crystallographic information

### 1. Comment

Bis-amine compounds are essential building blocks to produce branched or dendritic polymers. Dendrimers are an interesting class of materials which are based on bis-aromatic imine and amine precursors. These polymeric materials have attracted increasing attention due to their functional coordination groups, which can trap many metal ions or metal clusters within the voids in the dendrimers. This can lead to the formation of new types of organic-metallic hybrid nanomaterials (Kim *et al.*, 2005). Furthermore, the polyvalent nature of dendrimers is a key factor in generating a new class of drugs with much improved and acceptable pharmacokinetic profiles (Basavaraj *et al.*, 2009). This paper reports on a new addition to the bis-amine compounds and its chemical and physical features.

The compound, with a molar mass of 440.56 g mol^-1^, crystallizes in a monoclinic crystal structure with a space group notation of *P*2_1_/*n* and had a calculated density of 1.250 g cm^-3^. The asymmetric unit consists of half the molecule, the molecule is completed by inversion symmetry. Infrared spectra indicates typical absorbance bands of the functional phenyl group and amine –C=N group at 1570 and 1620 cm^-1^, respectively. The positive ES mass spectrum of the bis-amine showed a parent ion peak at m/*z* = 441.2362 (*M*+H)+, corresponding to C_32_H_28_N_2_, for which the required value = 440.2252.

### 2. Experimental

The bis-amine {N1,N4-dibenzhydrylbenzene-1,4-diamine} was prepared in a two-step procedure as follows: (i) A Schiff-base {N1,N4-bis-(diphenylmethylene)benzene-1,4-diamine} was synthesized by adopting a conventional procedure (Higuchi *et al.*, 2000) as follows: A mixture of benzophenone (1.69 g, 9.25 mmol), *p*-phenylenediamine (0.500 g, 4.62 mmol), and 1,4-diaza-bicyclo-[2.2.2]octane (DABCO) (3.11 g, 27.7 mmol) in chlorobenzene (40 ml) was stirred at room temperature for 10 min. Titanium (IV) tetrachloride (1.32 g, 6.93 mmol) dissolved in chlorobenzene (10 ml) was added dropwise using a pressure-equalized dropping funnel. The reaction mixture was heated in an oil bath at 125 °C for 24 h. The precipitate was removed by filtration, and then the filtrate was concentrated. The Schiff-base product (yield: 1.83 g, 91%) was isolated by silica gel uniplate chromatography with an eluent mixture of hexane:ethylacetate; 9:1, Rf = 0.25. (ii)The reduction of the Schiff-base was achieved using conventional procedures (Higuchi *et al.*, 2000; 2003) as follows: NaBH_4_ (0.06 g, 1.74 mmol) was added cautiously and in small portions to a mixture of the Schiff-base {N1,N4-bis-(diphenylmethyene)benzene-1,4-diamine} (0.500 g, 0.437 mmol), and SnCl_2_ (0.17 g, 0.87 mmol) dissolved in a mixture of dichloromethane/acetonitrile (1:1) (200 ml). The reaction mixture was stirred at room temperature for 10 min under an Argon atmosphere. The crude mixture was washed with an aqueous solution of 1% triethylamine (4x100), and the organic layer was dried over Na_2_SO_4_. The secondary bis-amine was purified from the crude product by uniplate silica gel chromatography with eluent (hexane: acetonitrile: chloroform; 8: 2: 1), Rf = 0.5. Yield: 0.98 g, 54.14%. Colourless plates were obtained from slow evaporation of a methanol solution of the bis-amine in air.

### 3. Refinement

Data were collected and processed according to Coles & Gale (2012). Hydrogen atoms were placed in geometrically calculated positions and included as part of a riding model with *U*_iso_ values set at 1.2 times *U*_eq_ of the parent atom.

### Crystal data

**Table:**

C_32_H_28_N_2_	*F*(000) = 468
*M**_r_* = 440.56	*D*_x_ = 1.250 Mg m^−^^3^
Monoclinic, *P*2_1_/*n*	Mo *K*α radiation, λ = 0.71075 Å
*a* = 14.784 (2) Å	Cell parameters from 5636 reflections
*b* = 5.5853 (8) Å	θ = 3.4–27.5°
*c* = 14.896 (2) Å	µ = 0.07 mm^−^^1^
β = 107.914 (8)°	*T* = 100 K
*V* = 1170.4 (3) Å^3^	Plate, colourless
*Z* = 2	0.1 × 0.09 × 0.02 mm

### Data collection

**Table:**

Rigaku AFC12 (Right) diffractometer	2664 independent reflections
Radiation source: Rotating Anode	1254 reflections with *I* > 2σ(*I*)
Confocal monochromator	*R*_int_ = 0.125
Detector resolution: 28.5714 pixels mm^-1^	θ_max_ = 27.5°, θ_min_ = 3.4°
profile data from ω–scans	*h* = −19→18
Absorption correction: multi-scan (*CrystalClear-SM Expert*; Rigaku, 2012)	*k* = −7→6
*T*_min_ = 0.345, *T*_max_ = 1.000	*l* = −16→19
10305 measured reflections	

### Refinement

**Table:**

Refinement on *F*^2^	Secondary atom site location: difference Fourier map
Least-squares matrix: full	Hydrogen site location: inferred from neighbouring sites
*R*[*F*^2^ > 2σ(*F*^2^)] = 0.077	H-atom parameters constrained
*wR*(*F*^2^) = 0.208	*w* = 1/[σ^2^(*F*_o_^2^) + (0.0886*P*)^2^] where *P* = (*F*_o_^2^ + 2*F*_c_^2^)/3
*S* = 0.97	(Δ/σ)_max_ < 0.001
2664 reflections	Δρ_max_ = 0.33 e Å^−^^3^
155 parameters	Δρ_min_ = −0.29 e Å^−^^3^
0 restraints	Extinction correction: *SHELXL97* (Sheldrick, 2008), Fc^*^=kFc[1+0.001xFc^2^λ^3^/sin(2θ)]^-1/4^
Primary atom site location: dual	Extinction coefficient: 0.026 (5)

### Special details

**Table:**

Experimental. FT—IR data were recorded on a Nicolet ATR FT—IR, while NMR data were collected on a Bruker 400 MHz s pectrometer in CD_2_Cl_2_– d2 solutions. The assignment of the chemical shifts for the NMR data were made following numbering shown in structure B. Schiff-base {N1,N4-bis(diphenylmethylene)benzene-1,4-diamine}IR (ATR cm-1) 1620 (C=N), 1597 and 1570 (phenyl). NMR data (p.p.m.), δH (400 MHz, CD2Cl2): 6.47 (4*H*, m; C3, 3`, 11, 11`-H), 7.06 (2*H*, d, J = 7.33 Hz; C15, 15`-H), 7.73 (2*H*, d, J = 7.33 Hz; C16, 16`-H), 7.27–7.40 (20*H*, m; aromatic-H); δC (100.63 MHz, CD2Cl2): 121.53–136.75, (aromatic carbon); 140.12 (C6, 6`,8, 8`); 147.37 (C14, 14`); 168.24 (C7, 7`); DEPT 13 C NMR exhibited no signals between 140–170 p.p.m.. The positive ES mass spectrum of the bis-amine showed the parent ion peak at m/*z* = 441.2362 (*M*+H)+ (95%) corresponding to C32H28N2; required value = 440.2252. Peaks detected at m/*z* =247.16 (100%) and 167.09 (98%), correspond to [*M*-(ph)2CH2)]+ and [*M*-(ph)2CH2+H2N2ph)]+, respectively.bis-amine {N1,N4-dibenzhydrylbenzene-1,4-diamine IR (ATR cm-1): 3392 (N—H), 2932; 2873 (C—H) aliphatic, 1599 and 1510 (phenyl). NMR data (p.p.m.), δH (400 MHz, CD2Cl2): 3.95 (2*H*, S, Na, a`-H), 5.36 (2*H*, S; C7, 7`-H), 6.37 (4*H*, d, J=7.33 Hz; C15, 15`, 16, 16`-H), 7.21–7.36 (20*H*, m, Ar—H); δC (100.63 MHz, CD2Cl2): 49.10 (C7, 7`); 115.21 (C15, 15`, 16, 16`); 127.25–129.04 (aromatic carbon); 140 (C6, 6`, 8, 8`); 144.07 (C14, 14`), DEPT 13 C NMR exhibited no signals between 140–145 p.p.m.. The positive ES mass spectrum of the bis-amine showed the parent ion peak at m/*z* = 441.2362 (*M*+H)+ (95%) corresponding to C32H28N2; required value = 440.2252. Peaks detected at m/*z* =247.16 (100%) and 167.09 (98%), correspond to [*M*-(ph)2CH2)]+ and [*M*-(ph)2CH2+H2N2ph)]+, respectively.
Geometry. All e.s.d.'s (except the e.s.d. in the dihedral angle between two l.s. planes) are estimated using the full covariance matrix. The cell e.s.d.'s are taken into account individually in the estimation of e.s.d.'s in distances, angles and torsion angles; correlations between e.s.d.'s in cell parameters are only used when they are defined by crystal symmetry. An approximate (isotropic) treatment of cell e.s.d.'s is used for estimating e.s.d.'s involving l.s. planes.
Refinement. Refinement of *F*^2^ against ALL reflections. The weighted *R*-factor *wR* and goodness of fit *S* are based on *F*^2^, conventional *R*-factors *R* are based on *F*, with *F* set to zero for negative *F*^2^. The threshold expression of *F*^2^ > σ(*F*^2^) is used only for calculating *R*-factors(gt) *etc*. and is not relevant to the choice of reflections for refinement. *R*-factors based on *F*^2^ are statistically about twice as large as those based on *F*, and *R*- factors based on ALL data will be even larger.

### Fractional atomic coordinates and isotropic or equivalent isotropic displacement parameters (Å^2^)

**Table:**

	*x*	*y*	*z*	*U*_iso_*/*U*_eq_	
N1	0.44891 (16)	0.4954 (4)	0.1672 (2)	0.0366 (7)	
H1	0.4144	0.6110	0.1765	0.044*	
C2	0.47809 (19)	0.3103 (5)	0.2375 (2)	0.0294 (8)	
H2	0.4635	0.1559	0.2050	0.035*	
C3	0.58440 (19)	0.3135 (5)	0.2909 (2)	0.0273 (7)	
C4	0.6426 (2)	0.5024 (5)	0.2829 (2)	0.0295 (8)	
H4	0.6169	0.6316	0.2440	0.035*	
C5	0.7386 (2)	0.4993 (5)	0.3323 (2)	0.0314 (8)	
H5	0.7768	0.6270	0.3265	0.038*	
C6	0.7785 (2)	0.3094 (5)	0.3901 (2)	0.0326 (8)	
H6	0.8431	0.3080	0.4231	0.039*	
C7	0.7208 (2)	0.1209 (5)	0.3983 (2)	0.0329 (8)	
H7	0.7468	−0.0081	0.4371	0.039*	
C8	0.6248 (2)	0.1228 (5)	0.3492 (2)	0.0325 (8)	
H8	0.5868	−0.0051	0.3554	0.039*	
C9	0.41899 (19)	0.3290 (5)	0.3050 (2)	0.0303 (8)	
C10	0.4294 (2)	0.5250 (5)	0.3644 (3)	0.0392 (9)	
H10	0.4723	0.6451	0.3626	0.047*	
C11	0.3771 (2)	0.5438 (6)	0.4259 (3)	0.0433 (9)	
H11	0.3845	0.6767	0.4651	0.052*	
C12	0.3137 (2)	0.3662 (6)	0.4296 (3)	0.0416 (9)	
H12	0.2782	0.3785	0.4712	0.050*	
C13	0.3032 (2)	0.1706 (6)	0.3712 (3)	0.0421 (10)	
H13	0.2614	0.0487	0.3742	0.051*	
C14	0.3545 (2)	0.1546 (5)	0.3081 (3)	0.0386 (9)	
H14	0.3454	0.0245	0.2673	0.046*	
C15	0.47494 (18)	0.4941 (5)	0.0834 (2)	0.0283 (8)	
C16	0.5290 (2)	0.3109 (5)	0.0627 (2)	0.0315 (8)	
H16	0.5489	0.1834	0.1042	0.038*	
C17	0.44676 (19)	0.6813 (5)	0.0199 (2)	0.0294 (8)	
H17	0.4107	0.8049	0.0332	0.035*	

### Atomic displacement parameters (Å^2^)

**Table:**

	*U*^11^	*U*^22^	*U*^33^	*U*^12^	*U*^13^	*U*^23^
N1	0.0298 (14)	0.0449 (16)	0.039 (2)	0.0124 (13)	0.0167 (14)	0.0079 (14)
C2	0.0222 (15)	0.0330 (16)	0.032 (2)	0.0024 (13)	0.0070 (14)	−0.0015 (14)
C3	0.0212 (14)	0.0322 (16)	0.030 (2)	−0.0007 (13)	0.0109 (14)	−0.0032 (14)
C4	0.0244 (15)	0.0290 (15)	0.035 (2)	0.0028 (13)	0.0094 (14)	0.0020 (14)
C5	0.0255 (15)	0.0340 (17)	0.035 (2)	−0.0023 (14)	0.0094 (15)	−0.0016 (15)
C6	0.0218 (15)	0.0415 (18)	0.034 (2)	0.0026 (14)	0.0076 (15)	−0.0063 (15)
C7	0.0279 (16)	0.0329 (17)	0.039 (2)	0.0084 (14)	0.0125 (16)	0.0026 (15)
C8	0.0257 (16)	0.0318 (17)	0.042 (2)	0.0006 (13)	0.0137 (16)	0.0046 (15)
C9	0.0210 (14)	0.0349 (17)	0.035 (2)	0.0010 (14)	0.0089 (14)	0.0025 (15)
C10	0.0319 (17)	0.0354 (18)	0.053 (3)	−0.0069 (15)	0.0177 (18)	−0.0066 (17)
C11	0.0378 (19)	0.047 (2)	0.048 (3)	0.0018 (17)	0.0177 (18)	−0.0068 (18)
C12	0.0345 (18)	0.050 (2)	0.048 (3)	0.0093 (17)	0.0248 (18)	0.0082 (18)
C13	0.0324 (18)	0.049 (2)	0.053 (3)	−0.0051 (16)	0.0253 (18)	0.0042 (18)
C14	0.0308 (17)	0.0357 (17)	0.053 (3)	−0.0058 (15)	0.0187 (17)	−0.0035 (16)
C15	0.0156 (13)	0.0345 (16)	0.032 (2)	−0.0022 (13)	0.0039 (14)	−0.0001 (15)
C16	0.0219 (14)	0.0351 (17)	0.037 (2)	0.0020 (13)	0.0086 (14)	0.0049 (15)
C17	0.0182 (14)	0.0350 (17)	0.036 (2)	0.0025 (13)	0.0106 (14)	−0.0022 (15)

### Geometric parameters (Å, º)

**Table:**

N1—H1	0.8600	C9—C10	1.386 (4)
N1—C2	1.440 (4)	C9—C14	1.373 (4)
N1—C15	1.415 (4)	C10—H10	0.9300
C2—H2	0.9800	C10—C11	1.372 (4)
C2—C3	1.528 (4)	C11—H11	0.9300
C2—C9	1.525 (4)	C11—C12	1.378 (4)
C3—C4	1.389 (4)	C12—H12	0.9300
C3—C8	1.388 (4)	C12—C13	1.375 (5)
C4—H4	0.9300	C13—H13	0.9300
C4—C5	1.384 (4)	C13—C14	1.380 (4)
C5—H5	0.9300	C14—H14	0.9300
C5—C6	1.379 (4)	C15—C16	1.391 (4)
C6—H6	0.9300	C15—C17	1.385 (4)
C6—C7	1.384 (4)	C16—H16	0.9300
C7—H7	0.9300	C16—C17^i^	1.384 (4)
C7—C8	1.382 (4)	C17—C16^i^	1.384 (4)
C8—H8	0.9300	C17—H17	0.9300
			
C2—N1—H1	118.8	C10—C9—C2	120.1 (2)
C15—N1—H1	118.8	C14—C9—C2	121.2 (3)
C15—N1—C2	122.4 (2)	C14—C9—C10	118.7 (3)
N1—C2—H2	107.6	C9—C10—H10	119.6
N1—C2—C3	113.6 (2)	C11—C10—C9	120.8 (3)
N1—C2—C9	109.0 (2)	C11—C10—H10	119.6
C3—C2—H2	107.6	C10—C11—H11	120.0
C9—C2—H2	107.6	C10—C11—C12	120.1 (3)
C9—C2—C3	111.2 (3)	C12—C11—H11	120.0
C4—C3—C2	121.9 (3)	C11—C12—H12	120.3
C8—C3—C2	119.5 (2)	C13—C12—C11	119.5 (3)
C8—C3—C4	118.6 (3)	C13—C12—H12	120.3
C3—C4—H4	119.8	C12—C13—H13	119.9
C5—C4—C3	120.4 (3)	C12—C13—C14	120.3 (3)
C5—C4—H4	119.8	C14—C13—H13	119.9
C4—C5—H5	119.6	C9—C14—C13	120.6 (3)
C6—C5—C4	120.9 (3)	C9—C14—H14	119.7
C6—C5—H5	119.6	C13—C14—H14	119.7
C5—C6—H6	120.6	C16—C15—N1	122.1 (3)
C5—C6—C7	118.9 (3)	C17—C15—N1	119.5 (3)
C7—C6—H6	120.6	C17—C15—C16	118.5 (3)
C6—C7—H7	119.7	C15—C16—H16	120.1
C8—C7—C6	120.6 (3)	C17^i^—C16—C15	119.9 (3)
C8—C7—H7	119.7	C17^i^—C16—H16	120.1
C3—C8—H8	119.7	C15—C17—H17	119.2
C7—C8—C3	120.7 (3)	C16^i^—C17—C15	121.7 (3)
C7—C8—H8	119.7	C16^i^—C17—H17	119.2
			
N1—C2—C3—C4	10.4 (4)	C4—C5—C6—C7	−0.2 (5)
N1—C2—C3—C8	−169.4 (3)	C5—C6—C7—C8	0.1 (4)
N1—C2—C9—C10	−67.2 (4)	C6—C7—C8—C3	0.0 (5)
N1—C2—C9—C14	112.5 (3)	C8—C3—C4—C5	−0.1 (4)
N1—C15—C16—C17^i^	−179.5 (3)	C9—C2—C3—C4	−113.0 (3)
N1—C15—C17—C16^i^	179.5 (3)	C9—C2—C3—C8	67.2 (3)
C2—N1—C15—C16	1.3 (4)	C9—C10—C11—C12	0.4 (5)
C2—N1—C15—C17	−178.1 (3)	C10—C9—C14—C13	−1.9 (5)
C2—C3—C4—C5	−180.0 (3)	C10—C11—C12—C13	0.0 (5)
C2—C3—C8—C7	179.9 (3)	C11—C12—C13—C14	−1.3 (5)
C2—C9—C10—C11	−179.7 (3)	C12—C13—C14—C9	2.3 (5)
C2—C9—C14—C13	178.4 (3)	C14—C9—C10—C11	0.6 (5)
C3—C2—C9—C10	58.8 (4)	C15—N1—C2—C3	69.6 (3)
C3—C2—C9—C14	−121.5 (3)	C15—N1—C2—C9	−165.8 (2)
C3—C4—C5—C6	0.2 (5)	C16—C15—C17—C16^i^	0.1 (5)
C4—C3—C8—C7	0.1 (4)	C17—C15—C16—C17^i^	−0.1 (4)

Symmetry code: (i) −*x*+1, −*y*+1, −*z*.
